# Short term outcomes following robotic arm-assisted lateral unicompartmental knee arthroplasty

**DOI:** 10.3389/fsurg.2023.1215280

**Published:** 2023-12-15

**Authors:** Warran Wignadasan, Justin Chang, Andreas Fontalis, Ricci Plastow, Fares S. Haddad

**Affiliations:** ^1^ Department of Trauma and Orthopaedic Surgery, University College Hospital, London, United Kingdom; ^2^ Department of Orthopaedic Surgery, Humber River Hospital, Toronto, ON, Canada; ^3^ Division of Orthopaedic Surgery, University of Toronto, Toronto, ON, Canada; ^4^ Department of Orthopaedic Surgery, The Princess Grace Hospital, London, United Kingdom

**Keywords:** lateral unicompartmental arthroplasty, MAKO arthroplasty, MAKO robot, lateral unicompartmental knee arthroplasty (LUKA), unicompartmental knee arthroplasty

## Abstract

**Introduction:**

Robotic-arm assisted medial unicompartmental knee arthroplasty (RA-UKA) is associated with improved accuracy of implant positioning and excellent early functional outcomes. However, there is paucity of evidence regarding outcomes following RA-UKA for isolated lateral compartment osteoarthritis. The purpose of this study was to assess the short-term clinical and patient reported outcomes of lateral compartment UKA, utilising robotic-arm assistance.

**Methods:**

This was a retrospective study of prospectively collected data of 21 consecutive patients who underwent lateral RA-UKA. The study included 9 (42.9%) males and 12 (57.1%) females with a mean age of 63.4 ± 9.2 years. The Oxford Knee Score (OKS) was measured pre-operatively and at 1-year post-operatively, while range of motion (ROM) and complications were also recorded.

**Results:**

There was significant improvement of OKS at 1 year's follow up compared with the baseline score (21.8 ± 5.6 vs. 45.2 ± 2.8 respectively; *p* < 0.001). There was also an improvement in pre-operative ROM when compared to ROM at 1 year's follow up (123.5° ± 8° vs. 131.5° ± 6.3° respectively; *p* < 0.001). None of the study patients underwent revision surgery within 1 year's follow-up.

**Conclusion:**

In our study, lateral RA-UKA resulted in significant improvements in clinical and patient reported outcomes with low complications rates. Further long-term comparative studies are needed to assess the utility of lateral RA-UKA vs. conventional UKA.

## Introduction

Total knee arthroplasty (TKA) represents a successful and effective form of treatment for patients suffering with end-stage arthritis of the knee (1). However, a significant proportion of these patients have disease, isolated to a single compartment of the knee. It has previously been reported that over 40% of patients who undergo TKA would be suitable for a unicompartmental knee arthroplasty (UKA) (2). UKA is less invasive than TKA with preservation of the unaffected compartments and ligamentous structures (3). There is strong evidence to suggest superior outcomes can be achieved compared to total knee arthroplasty (TKA) in relation to safety, cost-effectiveness and patient-reported outcomes, particularly in patients with severe comorbidities (1, 4–6). However, it is not without limitations; although there are fewer early post-operative complications (7, 8), UKA is associated with higher revision rates than TKA, yet it is not clear whether this is related to advancement of the disease or implant failure (9). Nonetheless, even when incorporating revision costs, UKA maintains a lower overall mean healthcare cost than TKA at 10 years post-operatively, accentuating its value in reducing the financial burden on healthcare systems in an era of an aging population (10).

Lateral compartment UKA is far less frequent compared to medial UKA owing to the medial compartment being associated with a higher incidence of osteoarthritis and osteonecrosis (11). Due to the anatomical and kinematic differences between the medial and lateral compartments, implants used for medial UKA may not be suitable for lateral UKA (12, 13). Moreover, the less constrained nature of the lateral compartment increases the technical complexity of performing a lateral UKA. The lateral flexion gap is greater than the extension gap, and there is increased lateral-sided laxity compared to the more constrained medial compartment (13–15). This increases the risk of bearing dislocation when mobile bearing implants are utilised (13, 16). However, advancement of surgical techniques and implant design have resulted in reduced rates of dislocations associated with lateral UKAs (17, 18). Due to the infrequency of lateral UKA being performed, there is a paucity of evidence on long-term survivorship, however mid-term results are very encouraging with studies reporting up to 100% survivorship (19, 20).

The evolution of surgical technology has resulted in the development of robotic-arm assistance, aiming to increase the precision and accuracy of bone cuts in order to enhance component alignment, optimise soft tissue balance, minimise surgical trauma and improve reproducibility of component placement (21–25). The use of a robotic arm for UKA has been shown to improve limb alignment and component positioning, reduce peri-operative pain, improve functional recovery, and reduce time to discharge compared with conventional jig-based knee arthroplasty (24, 26–31). The aim of this study was to assess clinical and patient reported outcomes following robotic-arm assisted lateral UKA (RA-UKA).

## Patients and methods

**Figure 1:**
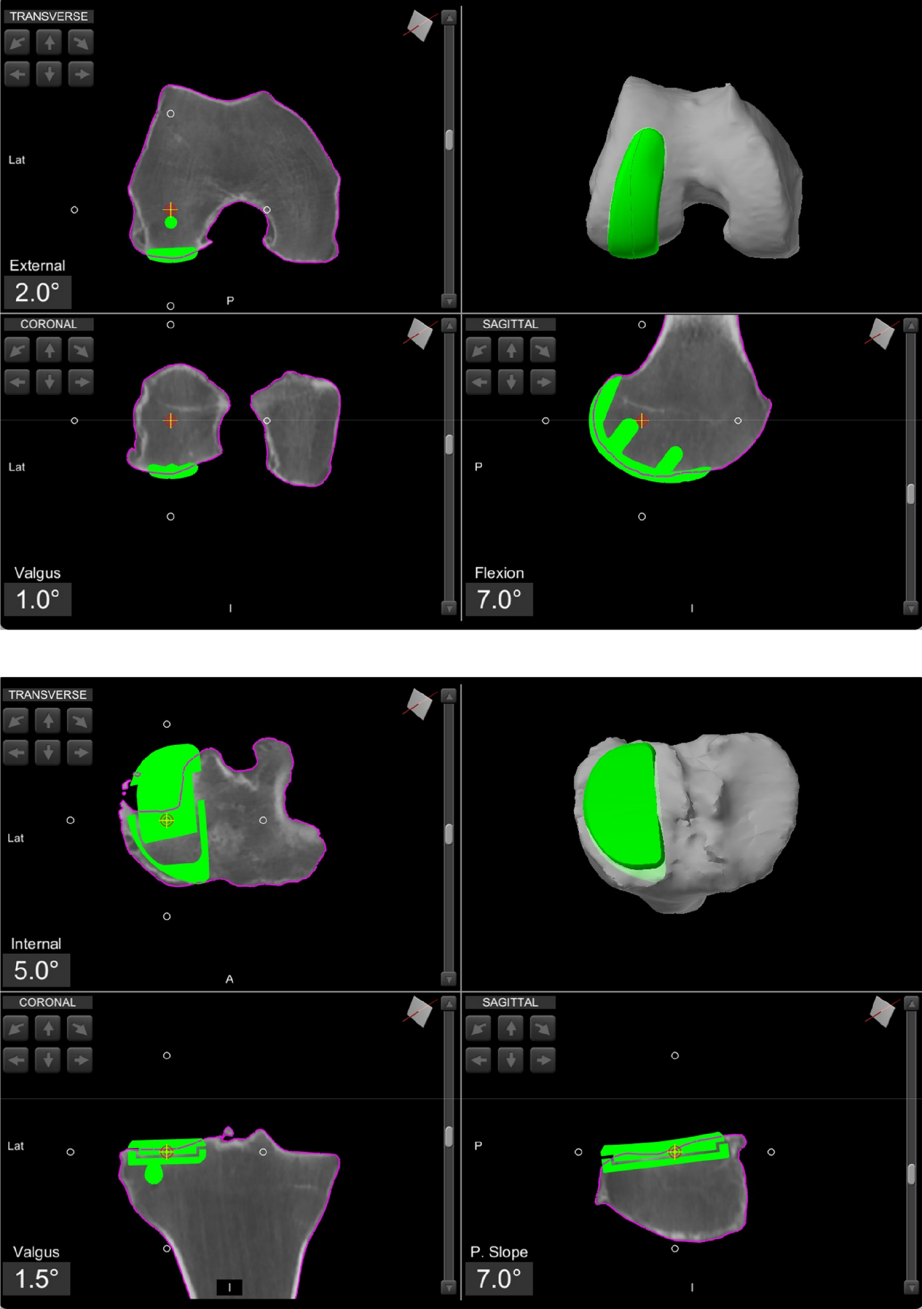
Pre-operative planning of a lateral UKA, using the robotic software.

A total of 21 patients that underwent lateral RA-UKA were included in this study. Data was retrospectively analysed from a prospectively collected database. All procedures were performed at a single tertiary referral centre. Inclusion criteria included all patients that underwent lateral RA-UKA with a minimum follow-up time of 1 year. All procedures were conducted using the MAKO Robotic Arm Interactive Orthopaedic System (Mako Surgical Corporation, Kalamazoo, MI, USA). The MAKO protocol involves the generation of a three-dimensional reconstruction model of the patient's knee, based on a pre-operative computer tomography (CT) scan. This model is then uploaded into the MAKO system's software, enabling the development of a personalised pre-operative plan and implant templating (Figure 1). All patients received the Restoris MCK Partial Knee implant (Mako Surgical Corporation, Kalamazoo, MI, USA). Patient demographic data included age, gender, body mass index (BMI) and American Society of Anaesthesiologists (ASA) grade. Patient reported and clinical outcomes were assessed using pre-operative and 1-year post-operative Oxford Knee Scores (OKS) and range of motion (ROM). Passive ROM was measured using a goniometer with the patient in a supine position.

### Surgical technique

**Figure 2:**
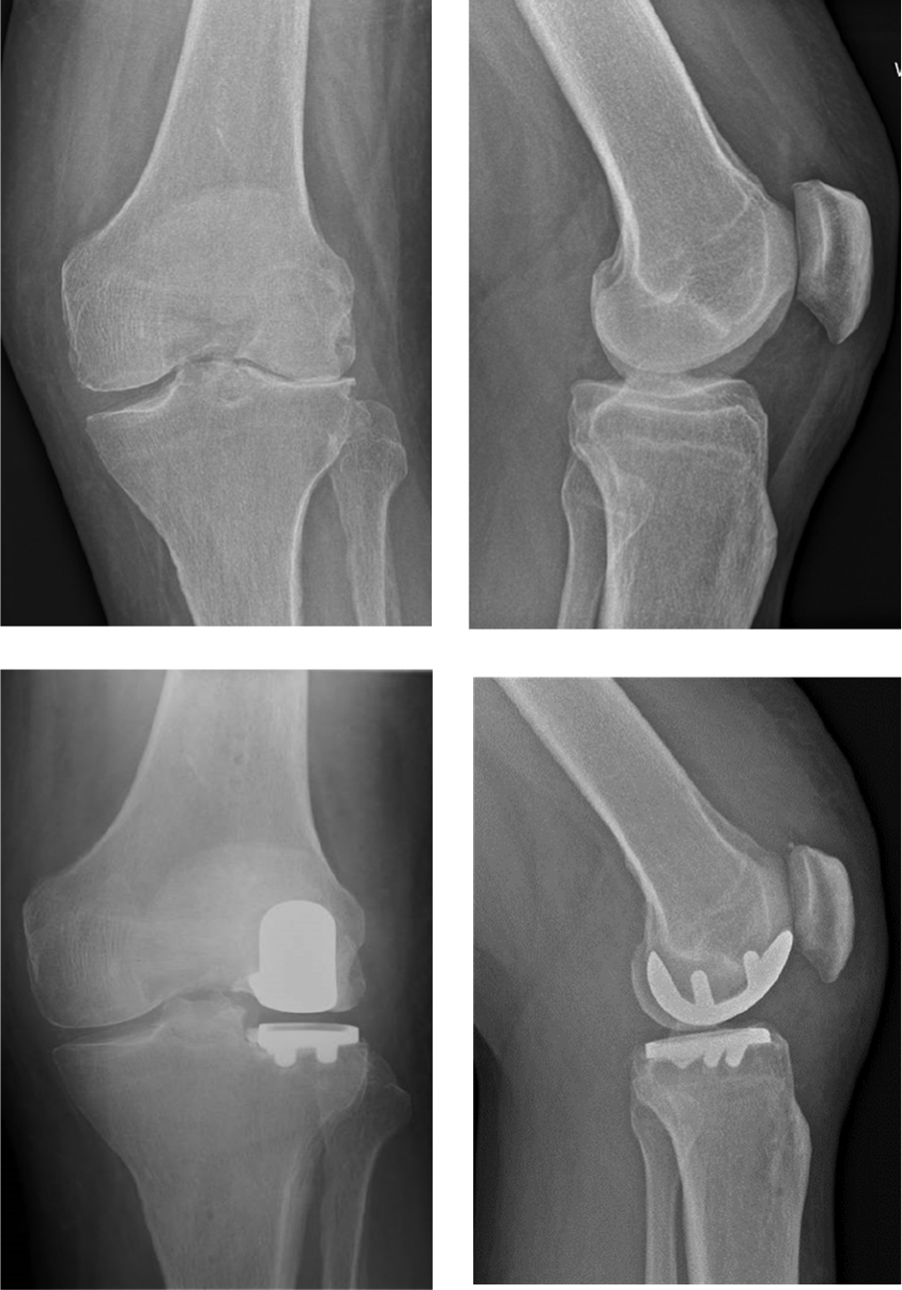
Pre- and post-operative radiographs depicting lateral UKA prosthesis.

All procedures were performed via a medial parapatellar approach with a medial arthrotomy that provides optimal access for the intra-incisional pin placement. This approach also allows access to other knee compartments and is preferred for revision surgery. Placement of the navigation pins for the femoral and tibial arrays were done within the original incision, which negated the necessity for separate incisions, potentially bearing further morbidity. Two bicortical femoral pins were placed anteromedially at the distal femoral shaft, avoiding the anterior aspect of the femoral component. The two bicortical tibial reference pins were placed at the level of the tibial tubercle, distal to the tibial tray. Following array placement, enabling intra-operative measurement of the alignment and kinematics; bone registration was performed by mapping knee landmarks displayed on the robotic system's screen. To achieve optimal ligament balancing, the knee was positioned in a comfortable position in space, partially correcting the valgus deformity while being mindful of avoiding overcorrection. We aimed to achieve lower limb alignment in the individual's constitutional alignment. Through careful observation of kinematics alignment and visualization of tracking, adjustments were made to the plan as needed in order to attain the desired alignment. Bone resection was performed within haptic boundaries using a high-speed oscillating saw and a burr. Optical motion capture technology was utilised for the assessment of flexion-extension gaps, limb alignment, ligamentous laxity and ROM with trial implants inserted. Once the implants had been chosen based on pre-operative planning and intra-operative trialling, the definitive fixed bearing Restoris MCK implants were cemented (Figure 2).

## Results

### Baseline characteristics

Twenty-one patients who underwent lateral RA-UKA and had minimum 1 year follow-up were included in the study. There were 9 (42.9%) males and 12 (57.1%) females. The mean age of the study cohort was 63.4 ± 9.2 years (range 51–80 years). Mean BMI was 32.4 ± 4.3. Two thirds of the study participants were classified as ASA II, while nearly a quarter were classified as ASA III (Table 1). Mean pre-operative valgus alignment measured on AP radiographs was 8.8 ± 2.6° (range 5–15°) (Table 1).

**Table 1:** Baseline characteristics in patients undergoing robotic-arm assisted UKA.

Characteristics	Robotic-arm assisted UKA (*N* = 21)
Age (years)	63.4 ± 9.2
Body mass index (Kg/m^2^)	32.4 ± 4.3
Gender	
Female	12 (57.1%)
Male	9 (42.9%)
ASA class	
I	2 (9.5%)
II	14 (66.7%)
III	5 (23.8%)
Pre-operative valgus alignment (degrees)	8.8 ± 2.6

ASA, American society of anesthesiologists score; UKA, unicompartmental knee arthroplasty.

The values are given as the mean ± standard deviation or as the number with the percentage in parentheses.

### Patient reported outcome measurements (PROMS) and range of motion (ROM)

**Figure 3:**
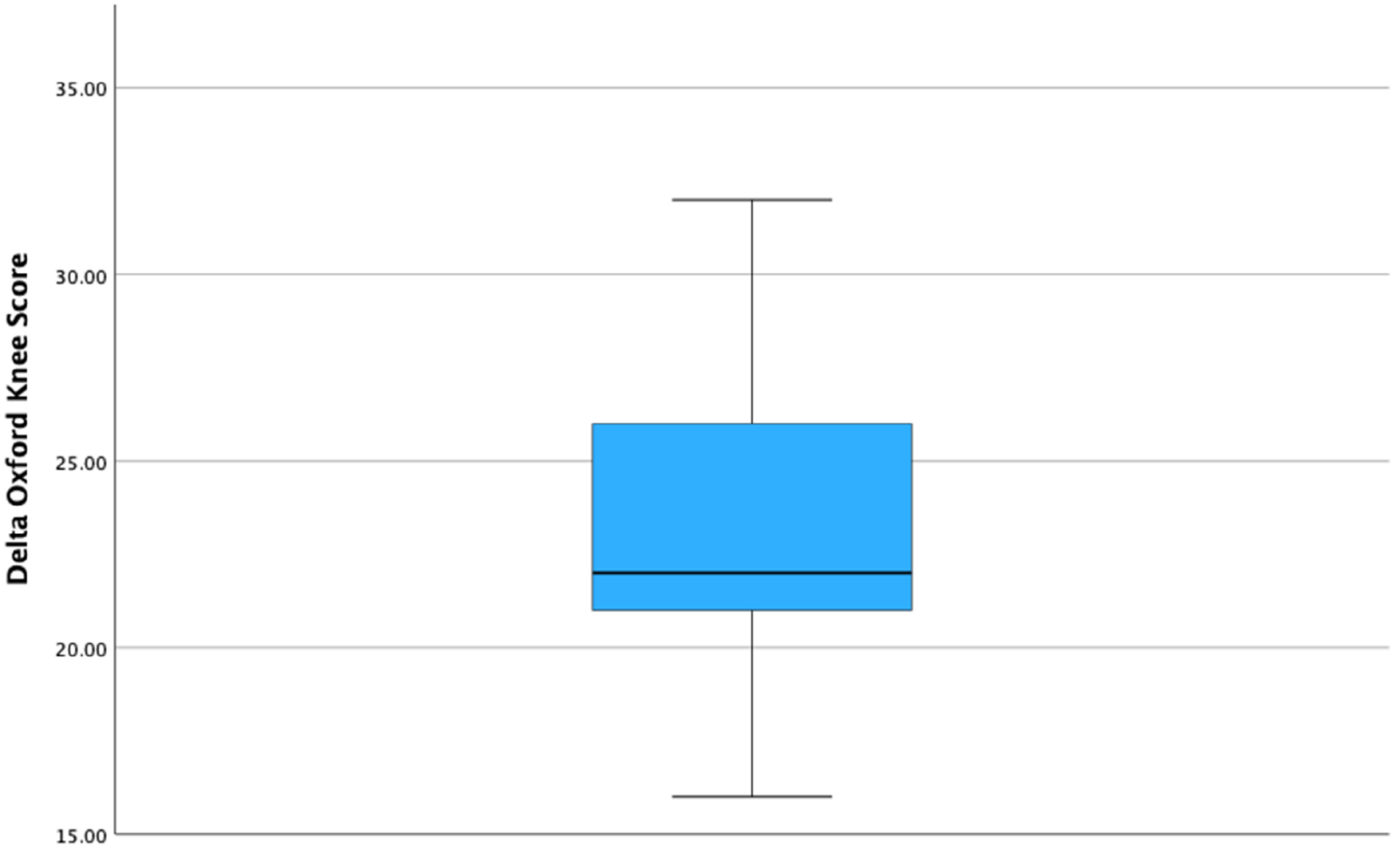
Boxplot, illustrating the delta Oxford knee score among patient undergoing robotic-arm assisted UKA.

**Figure 4:**
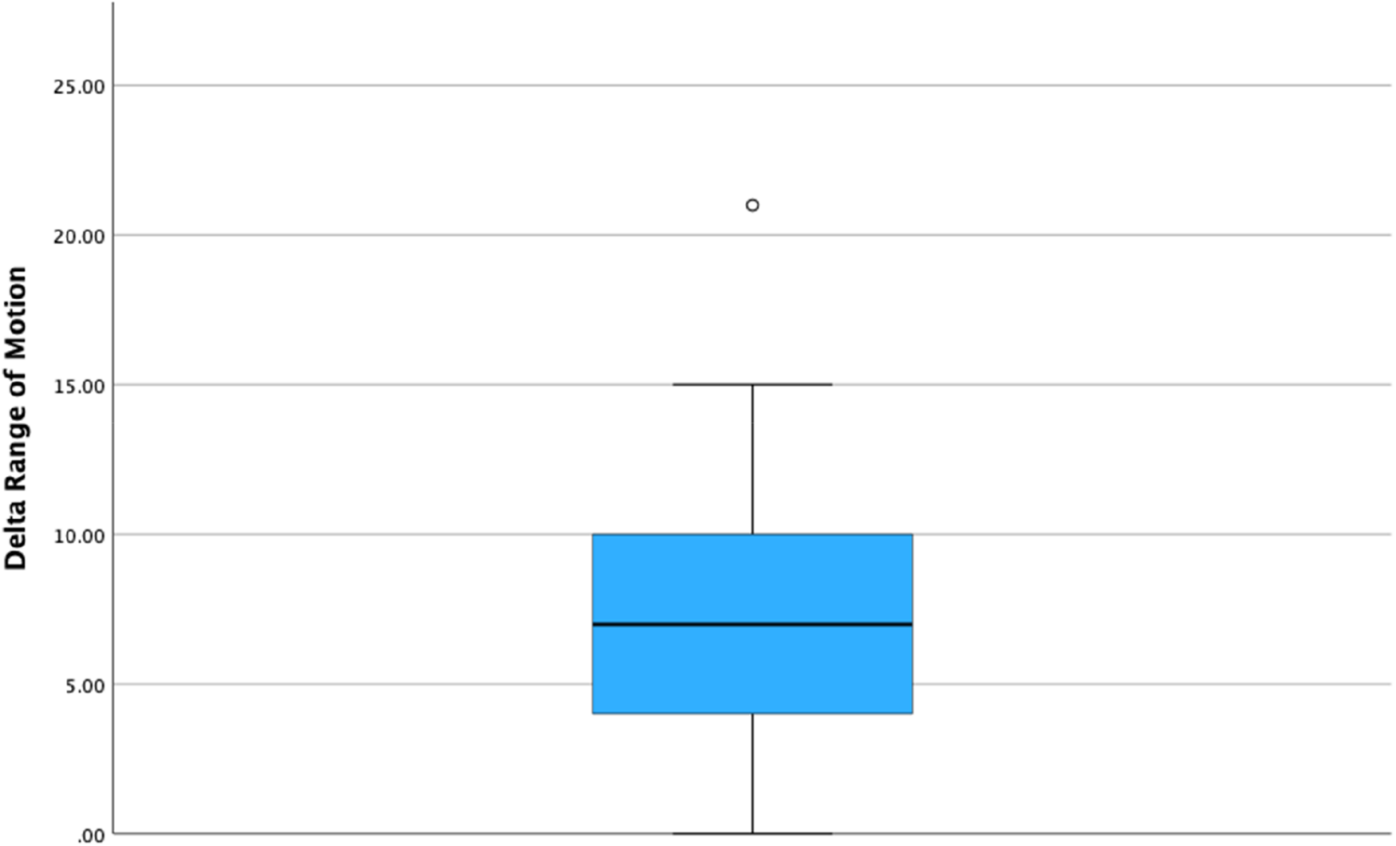
Figure illustrating the delta range of motion among patient undergoing roboticarm assisted UKA.

Lateral RA-UKA was associated with a statistically significant improvement in PROMs as measured by the OKS at 1 year's follow up (21.8 ± 5.6 pre-operatively vs. 45.2 ± 2.8 at 1 year's follow up, *p* < 0.001). The mean difference in OKS during the study was 23.4 ± 4.2. Figure 3 illustrates a box plot of the delta-OKS values. A statistically significant improvement in ROM post-operatively was also evident at the 1-year post-operative timepoint (mean pre-operative flexion of 123.5° ± 8° vs. 131.5° ± 6.3° at 1 year follow up; *p* < 0.001) (Table 2). Mean delta-ROM was 8° ± 5° (Figure 4).

**Table 2:** Table depicting patient reported outcome measures and range of motion pre-operatively and at one year follow-up.

Variables	Robotic-arm assisted UKA (*N* = 21)	*P* value
Oxford knee score (OKS)		
Baseline	21.8 ± 5.6	
1-year's follow up	45.2 ± 2.8	<0.001^a^
Delta—OKS	23.4 ± 4.2	
Range of motion (ROM)		
Baseline	123.5 ± 8°	
1-year's follow up	131.5 ± 6.3°	<0.001^a^
Delta-ROM	8 ± 5°	

Data presented as mean ± standard deviation.

^a^ Paired-samples *T*-test.

**Figure 5:**
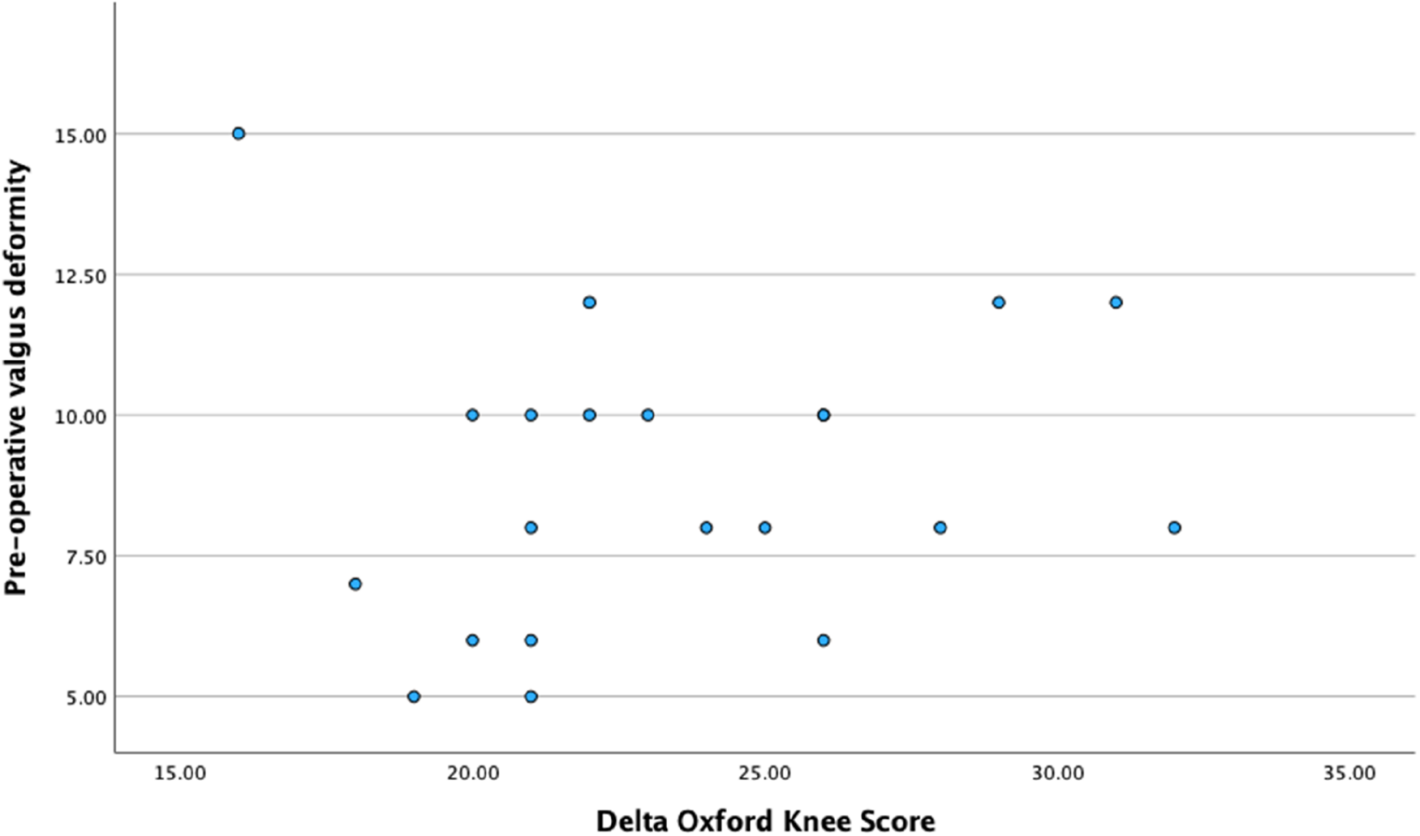
Scatter plot illustrating the relationship between pre-operative valgus deformity and delta Oxford knee score.

**Figure 6:**
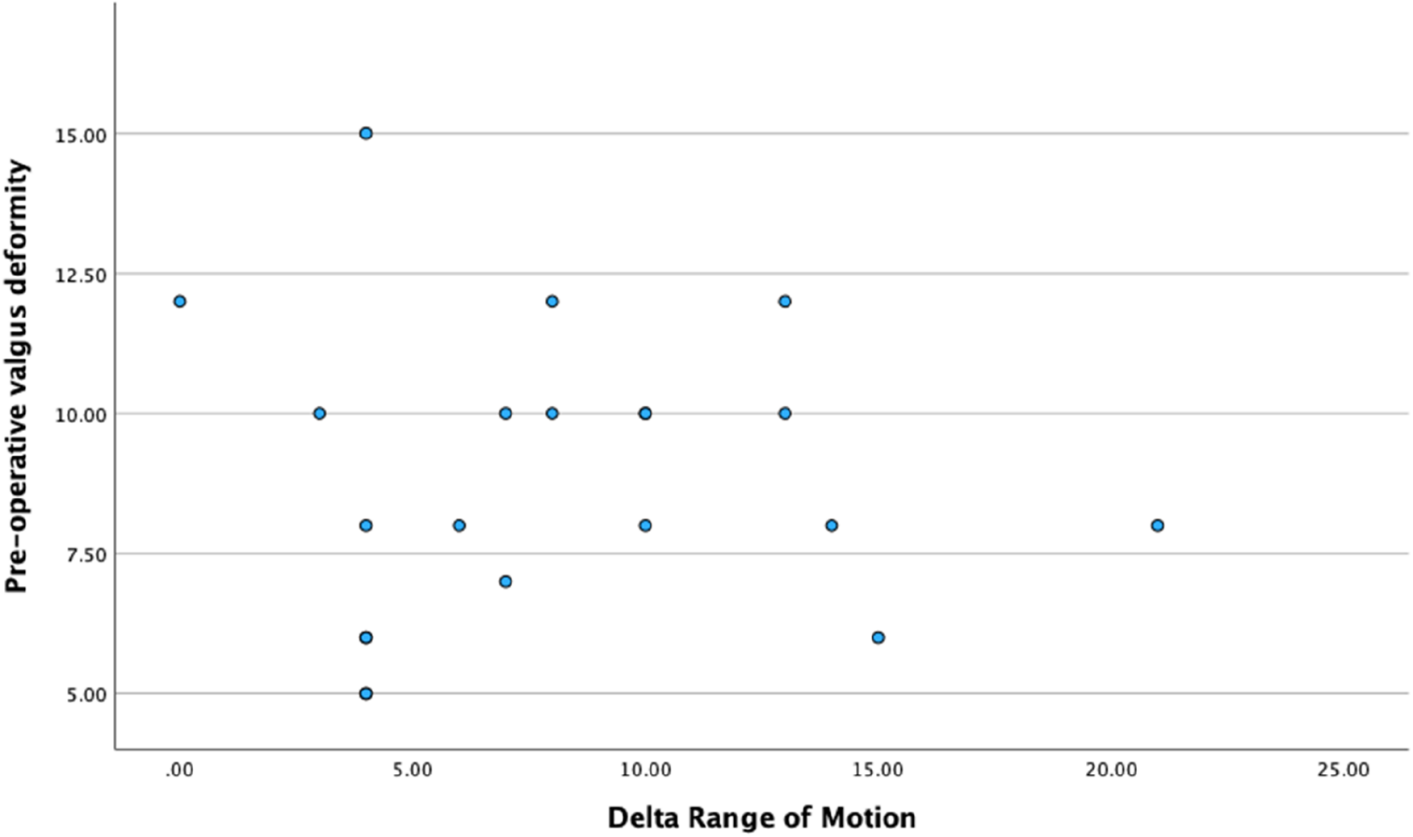
Scatter plot illustrating the relationship between pre-operative valgus deformity and delta range of motion.

**Figure 7:**
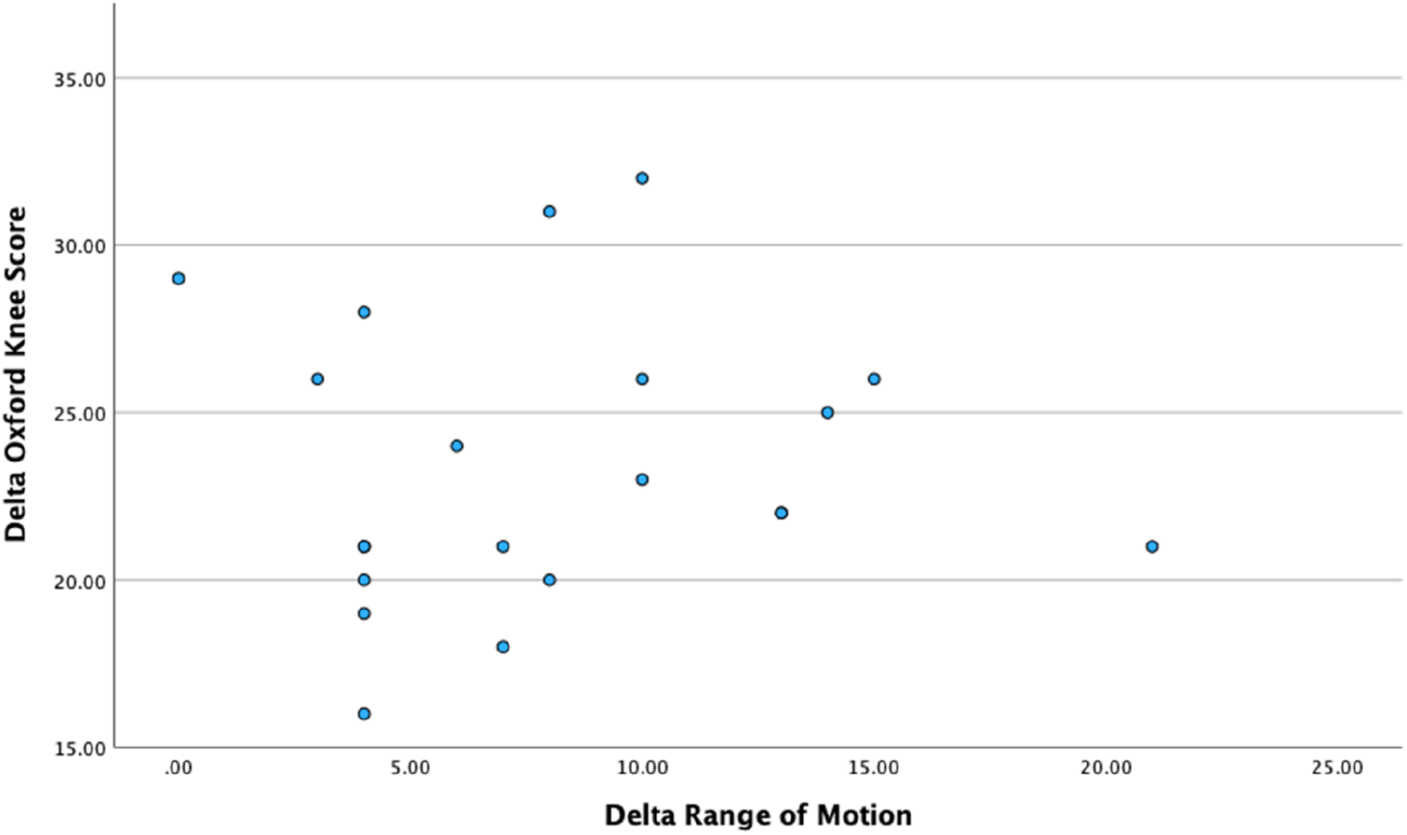
Scatter plot illustrating the relationship between delta range of motion and delta Oxford knee score.

Correlational analyses did not reveal any significant relationship between pre-operative valgus deformity and Delta-OKS or Delta-ROM (Table 3). Furthermore, no significant correlation was evident between the delta-ROM and delta-OKS (Table 3 and Figures 5–7 illustrate).

**Table 3:** Correlation among delta Oxford knee score, range of motion and pre-operative valgus deformity.

Variables	Pre-operative valgus deformity	Delta Oxford knee score
Delta Oxford knee score (OKS)	0.104 (*p* = 0.653)^b^	
	0.248 (*p* = 0.278)^a^	
Delta range of motion (OKS)	−0.30 (*p* = 0.898)^b^	0.34 (0.883)^b^
	0.059 (*p* = 0.8)^a^	0.142 (0.54)^a^

^a^ Spearman correlation coefficient.

^b^ Pearson correlation coefficient.

### Ligament balance in flexion and extension

**Figure 8:**
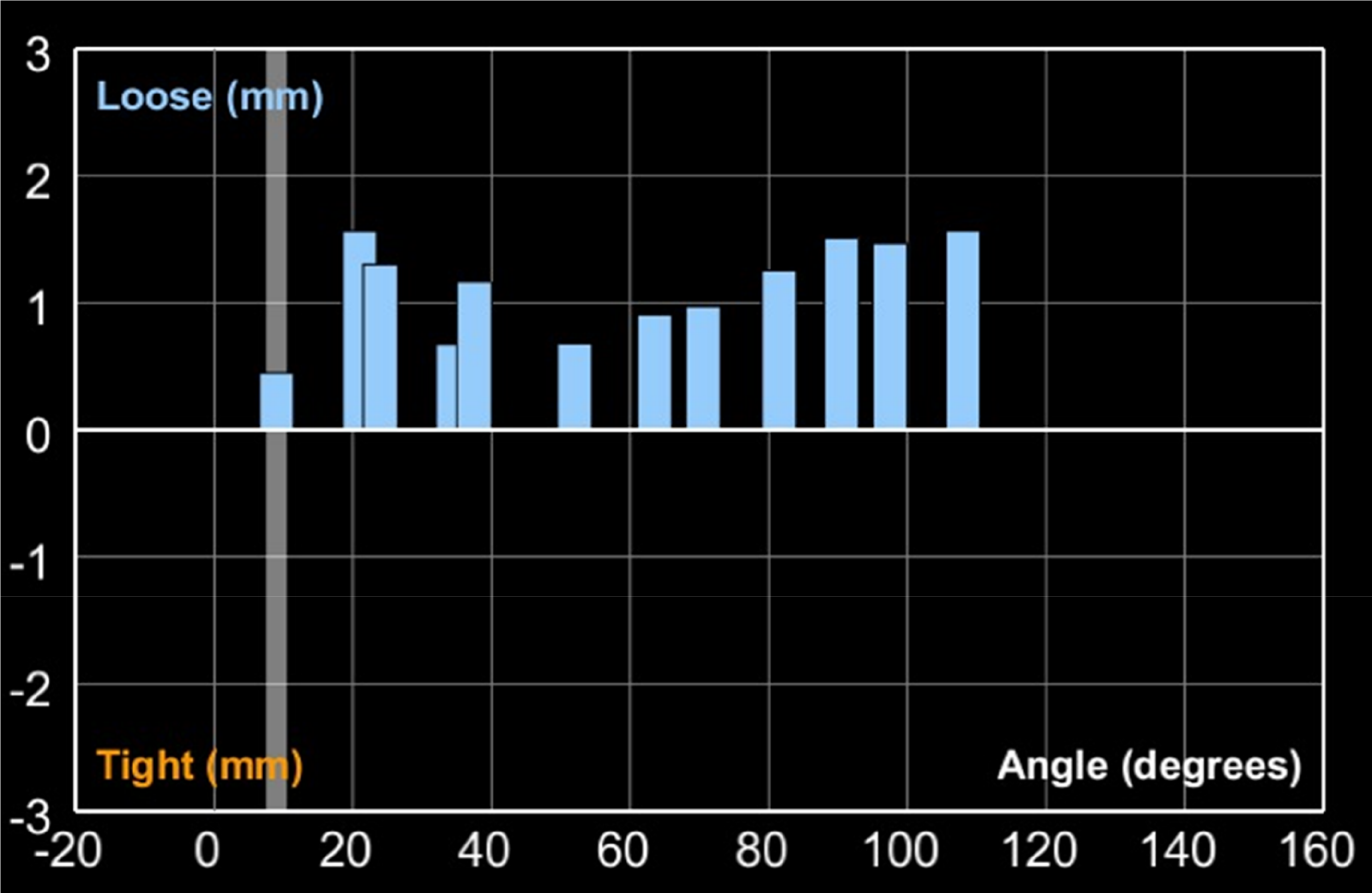
Graph showing balancing curve intra-operatively across the knee range of motion.

Following the application of varus stress to bring the knee into its native alignment, ligament balancing was assessed across various flexion-extension cycles. The robotic system provides real-time feedback regarding ligament balancing, depicted as the deviation in millimetres (mm) from the optimal tracking of the prosthesis at different angles. Negative values signify ligamentous tightness and positive values represent laxity. Figure 8 depicts the intra-operative balancing graph for one of the study's participants, spanning from full extension to beyond 90° of flexion. Among the 21 patients in this study, we noted the mean laxity in extension was 0.5 ± 0.4 mm; at 30° flexion 0.8 ± 0.4 mm; at 60° flexion 1.3 ± 0.6 mm; and at 90° flexion 1.6 ± 0.7 mm.

### Complications

None of the patients in our study cohort required revision surgery. One patient developed a superficial infection post-operatively which resolved following oral antibiotic treatment. No other complications or issues were recorded during the recovery phase or at the post-operative follow up appointments.

## Discussion

Lateral UKA is a more demanding and technically challenging procedure compared to medial UKA. The introduction of robotic-arm assistance provides the opportunity to create an elaborate and personalised pre-operative plan, in addition to evaluating knee kinematics and alignment intra-operatively. Given the complexities of UKA and potential consequence of errors, the aim of our study was to assess the clinical and patient reported outcomes utilising robotic-arm assistance. We found that lateral RA-UKA resulted in significant improvement of functional outcomes, using ROM and OKS as surrogate measures.

Our findings are in concordance with previous studies. Burger et al., in their study encompassing 171 lateral RA-UKAs, reported an improvement in Knee Injury and Osteoarthritis Outcome Scores (KOOS) and excellent mid-term survivorship (32). Authors also reported a mean Forgotten Joint Score (FJS) of 81 at 2 years' follow up. Excellent KOOS scores were also reported by another study, with a mean improvement of 54 points at 3 years following lateral RA-UKA (33). This study also reported superior mean FJS scores (85.1) (33). Canetti et al. studied return to sporting activity between patients undergoing lateral RA-UKA compared with lateral UKA using the conventional technique at mean follow up of just over 3 years (34). Authors reported that lateral RA-UKA was associated with a significantly quicker return to sports compared with the conventional technique (4.2 ± 1.8 months vs. 10.5 ± 6.7 months). Favourable outcomes with the utilisation of robotic-arm assistance in lateral UKA have been reported by several other studies (35–40), showcasing the positive impact of precise bone cuts and accurate implant positioning.

With respect to correlational analyses, no significant relationship was illustrated between pre-operative valgus deformity, Delta-ROM and Delta-OKS. A possible explanation could involve our small sample size. However, an alternative interpretation could embrace the fact that robotic arm-assistance consistently led to significant improvement in PROMs and functional outcomes. This highlights that the severity of the pre-operative valgus deformity did not influence improvements seen in ROM and PROMs, thus emphasising the reproducibility of outcomes using robotic technology. Heckmann et al. evaluated the mid-term outcomes of 84 patients who underwent lateral RA-UKA and also found no correlation between degree of correction and PROMs in their study (36), further highlighting the reliability and reproducibility of using a robotic arm. Interestingly, this study also included four patients with well-controlled inflammatory arthritis without any reported complications.

Van der List et al. assessed PROMs in patients undergoing medial (143 knees) vs. lateral (36 knees) RA-UKA and found no significant differences in Western Ontario and McMaster University Arthritis (WOMAC) scores or FJS (37). Interestingly, authors reported that optimal alignment differed between the two groups. Patients in the medial RA-UKA group in whom neutral alignment was achieved (−1° to −3°), demonstrated superior FJS. Whereas, in the lateral RA-UKA cohort, superior FJS and WOMAC scores were seen with slight under-correction (3°–7°) (37). Khamsaisy et al. found that there was an increased risk of overcorrection as well as a greater difficulty in predicting post-operative alignment when performing lateral RA-UKA compared to medial RA-UKA (35).The intrinsic laxity of the lateral compartment may explain the increased risk of overcorrection during lateral UKA, highlighting the importance of being mindful of the soft tissue differences between the medial and lateral compartments when performing lateral UKA.

Although our study reports on the short-term results of lateral RA-UKA, studies in the literature have portrayed positive results with regards to longer-term survivorship of lateral RA-UKA. Zambianchi et al. reported 100% survivorship of the 67 lateral RA-UKA performed with mean follow up of 36.3 months (33). Thein et al. and Batallier et al. also reported 100% survivorship in their short-term studies analysing lateral RA-UKA (39, 41). A systematic review evaluating mid-term follow-up of RA-UKA (which included seven studies that analysed lateral RA-UKA) reported a survivorship of 96% (26). There were no studies reporting on long-term survivorship of lateral RA-UKA owing to the relative novelty of robotic technology combined with the infrequency that lateral UKA is performed.

Our study has limitations. Firstly, our cohort size is relatively small and a comparator group was not applicable. Moreover, this is a series performed by a high-volume surgeon (senior author) at centres where the use of the robotic arm has increased significantly over the preceding years. However, given the short learning curve with robotic-arm assistance, we believe our findings could be generalisable to lower-volume institutions. Further studies are needed to evaluate longer-term outcomes and survivorship, in addition to ascertaining the reproducibility of excellent outcomes by less experienced surgeons.

In conclusion, the use of robotic arm-assistance for lateral UKA was shown to be associated with a significant improvement in clinical outcomes, irrespective of the severity of the pre-operative deformity, with low complication and reoperation rates. Further multi-centre comparative studies assessing longer-term outcomes and survivorship of lateral RA-UKA are needed.

## Data Availability

The data that support the conclusions of this article are available to other researchers by the authors, upon reasonable request.
